# Case report: IgG4-related mass-forming thyroiditis accompanied by regional lymphadenopathy

**DOI:** 10.1186/s13000-017-0681-9

**Published:** 2018-01-03

**Authors:** Yasuhiro Sakai, Yoshiaki Imamura

**Affiliations:** 1 Department of Tumor Pathology, Faculty of Medical Sciences, University of Fukui, 23-3 Matsuoka-Shimoaizuki, Eiheiji, Fukui, 910-1193 Japan; 2 Division of Diagnostic Pathology/Surgical Pathology, University of Fukui Hospital, Eiheiji, Japan

**Keywords:** IgG4-related disease, Mass-forming thyroiditis, Regional lymphadenopathy

## Abstract

**Background:**

It has been recently accepted that IgG4-related thyroiditis is comparable to the Hashimoto and Riedel thyroiditis and Graves disease which are rich in IgG4-secreting plasma cells. Many physicians believe that in IgG4-related thyroiditis, the thyroid is entirely enlarged and diffusely affected, which is similar to conventional thyroiditis, but rarely ever accompanied by pseudoneoplastic mass formation as in IgG4-related disease in the other organs. This report introduces another pattern of IgG4-related thyroiditis as mass-forming thyroiditis and presents the occurrence of IgG4-related regional lymphadenopathy as an unusual accompanying symptom.

**Case presentation:**

A 66-year-old woman presented with an approximately 2.5-cm mass in the right thyroidal lobe and regional lymph node swelling, which were preoperatively misinterpreted as thyroidal carcinoma. After lobectomy, histological examination was performed, revealing that the mass showed dense stromal fibrosis, lymphoplasmacytic infiltration, and effacement of thyroid follicles, while the background thyroidal tissue seemed to mimic lymphocytic thyroiditis without fibrosis. Immunohistochemistry revealed predominance of IgG4-secreting plasma cells among infiltrating lymphocytes independent of mass lesion or background tissue. In addition, the regional Delphian and paratracheal lymph nodes were swollen, histologically showing numerous IgG4-secreting plasma cell infiltrations in the interfollicular zone.

**Conclusions:**

IgG4-related mass-forming thyroiditis, which may be an extremely rare but recognizable pattern of IgG4-related thyroiditis, may be distinguishable from Hashimoto and Riedel thyroiditis, Graves disease, and thyroidal carcinoma. In addition, the regional IgG4-related lymphadenopathy, also possibly misdiagnosed as metastatic thyroidal carcinoma, may be a newly recognized manifestation of IgG4-related thyroiditis.

## Background

IgG4-related disease is a spectrum of inflammatory disorders characterized by lymphocyte infiltrates that are rich in IgG4-secreting plasma cells [1]. It is histologically characterized by lymphocyte and plasma cell infiltration and storiform fibrosis, and is usually associated with obliterative phlebitis and increased serum IgG4 level [1, 2]. Because IgG4-related disease is more widely recognized as a fibroinflammatory disorder mediated by immune abnormality, some inflammatory disorders once considered to involve a single organ are now recategorized into the IgG4-related disease spectrum, such as Mikulicz disease of the salivary gland [3], autoimmune pancreatitis [4], idiopathic retroperitoneal fibrosis [5], and a subset of inflammatory pseudotumors and pseudolymphomas [6–8]. Recently, some cases of Hashimoto and Riedel thyroiditis have also been included in IgG4-related disease of the thyroid [9–11]. However, unlike IgG4-related diseases of the other organs, IgG4-related Hashimoto and Riedel thyroiditis rarely ever form a pseudoneoplastic fibrous mass and often cause diffuse thyroid swelling, like in non-IgG4-related thyroiditis [12]. Herein, we present the case of IgG4-related mass-forming thyroiditis that differed from typical Hashimoto and Riedel thyroiditis. In addition, we also report an interesting finding of IgG4-related regional lymphadenopathy that accompanied the IgG4-related mass-forming thyroiditis.

## Case presentation

### Clinical history

A 66-year-old Japanese woman presented with a 5-month history of cough and sore throat. Clinical examination revealed a palpable elastic hard mass on the right side of the neck; ultrasonography and computed tomography revealed the mass to be in the lower pole of the right thyroidal lobe without extrathyroidal extension and the Delphian and paratracheal lymph nodes to be slightly enlarged. No other enlarged lymph nodes and metastatic lesions were detected in the body. Serum thyroid stimulating hormone (29.32 μIU/mL, reference range: 0.35–4.94 μIU/mL) and anti-thyroid peroxidase antibody (576 IU/mL, reference range: 0–16 IU/mL) levels were elevated. Serum free T3 (1.42 pg/mL, reference range: 1.71–3.71 pg/mL) and serum free T4 (0.46 ng/dL, reference range: 0.70–1.48 ng/dL) levels were slightly decreased; however, other laboratory data were normal, including thyroglobulin (1.73 ng/mL, reference range: 0–33.7 ng/mL) and IgG4 (60.3 mg/dL, reference range: 4.8–105 mg/dL) levels. Fine needle aspiration of the thyroidal mass obtained follicular cell clusters containing less amounts of colloid, which were categorized as “atypia of undetermined significance or follicular lesion of undetermined significance (AUS/FLUS),” in a background slightly rich in lymphocytes and plasma cells [13]. The patient and her family had no relevant previous history of disease. The clinical and radiological findings indicated thyroidal cancer rather than thyroidal inflammatory disorders such as subacute and Hashimoto thyroiditis; therefore, partial thyroidectomy (right lobectomy) with Delphian and paratracheal lymph node dissection was carried out after careful informed consent. The postoperative course was uneventful.

### Thyroid histology

**Fig. 1:**
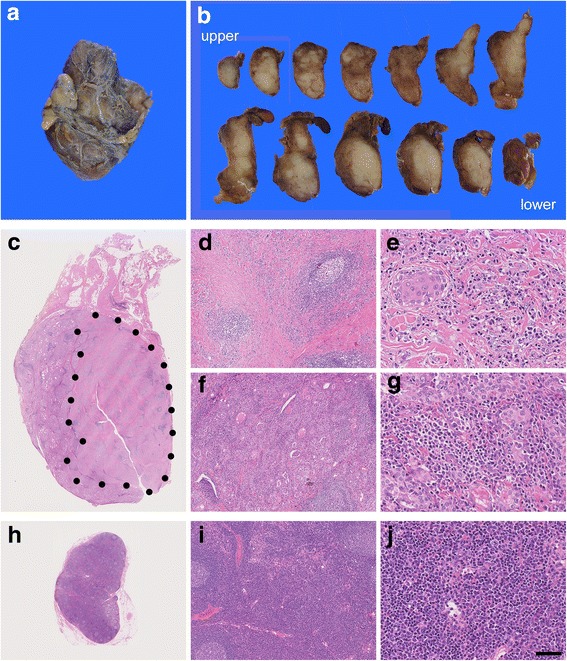
Gross and histological findings. **a**, **b** A circumscribed whitish mass measuring approximately 2.5 cm located in the lower pole of the right thyroidal lobe was misdiagnosed as thyroidal carcinoma after clinical and radiological examination. Another sclerotic lesion in the upper pole had not been detected clinically. **c** An intrathyroidal sclerotic mass was formed (dotted area). **d**, **e** Histologically, the mass lesion was composed of dense stromal fibrosis and lymphoplasmacytic infiltration with occasional lymphoid follicles. Thyroid follicles were almost effaced, and squamous metaplasia (morulae) was sparsely observed. **f**, **g** Background thyroidal tissue was reminiscent of lymphocytic thyroiditis; however, plasma cells were more richly infiltrated. **h**–**j** Regional lymph nodes were slightly enlarged. Histologically, the interfollicular zone contained numerous plasma cells as well as small lymphocytes and eosinophils. Bar = 1 cm for **a** and **b**; 2.5 mm for **c** and **h**; 250 μm for **d**, **f** and **i**; and 50 μm for **e**, **g** and **j**

Gross examination revealed that whitish, firm, somewhat nodular lesions were distributed into the upper and lower poles (Fig. 1a and b). Particularly in the lower pole, lesions were fused, forming a somewhat circumscribed mass measuring approximately 2.5 cm in diameter, which was clinically and radiologically misinterpreted as thyroidal cancer.

As shown in Fig. 1c–g, the histology seems to differ between mass lesion and background thyroidal tissue. In the lower pole’s mass, interstitial storiform fibrosis extended abundantly; however, it did not extend beyond the thyroid capsule. Most of the follicular epithelium was effaced, and a few cells underwent squamous metaplasia and formed morulae sporadically. There was extensive infiltration of lymphocytes and plasma cells with the occasional formation of well-developed germinal centers. Meanwhile, in the background tissue, lymphocytes and plasma cells intensely infiltrated the parenchyma, as seen in lymphocytic thyroiditis; however, storiform fibrosis did not occur. The thyroid follicles were not effaced but atrophic and regenerative with less colloid. Obstructive and non-obstructive phlebitis was not observed.

**Fig. 2:**
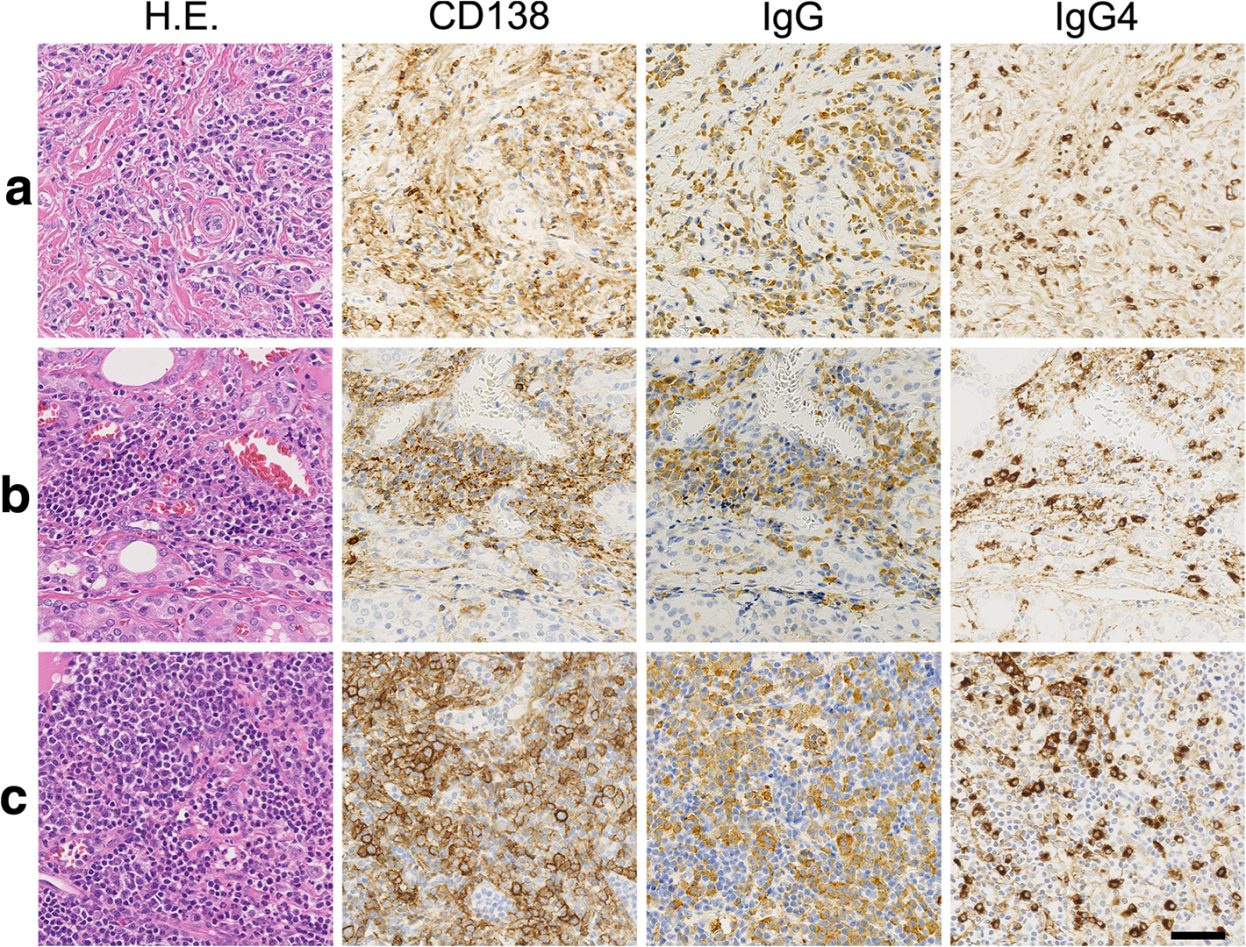
Immunohistochemical findings of (**a**) lower pole’s mass, (**b**) background tissue in the thyroid, and (**c**) the regional lymph node. Numerous CD138^+^ plasma cells were infiltrated in the lower pole’s mass, background of thyroidal tissue, and regional lymph nodes. Most were IgG-secreting plasma cells, and 40–50% of them were IgG4-secreting cells. Bar = 50 μm

As shown in Fig. 2a and b, immunohistochemical analysis revealed that the infiltrating lymphocytes included numerous IgG4-secreting plasma cells (45–55 cells/high power field), and the IgG4/IgG-secreting plasma cell ratio was increased (approximately 49.4%) in both the whitish sclerotic nodular lesions and the background thyroidal tissue. In-situ hybridization assay demonstrated that the κ and λ light-chain-producing plasma cell populations did not differ significantly.

### Regional lymph nodes

The Delphian and paratracheal lymph nodes were grossly swollen, measuring 7 mm and 11 mm, respectively (major axis). Histologically, lymph node architecture was reactively hyperplastic, and plasma cells, including IgG4-secreting cells, were infiltrated in great number, particularly in the interfollicular area of the inner cortex (Figs. 1h–j and 2c).

## Discussion

IgG4-related disease is a spectrum of inflammatory disorders characterized by lymphocyte infiltrates dominated by IgG4-secreting plasma cells [1, 2]. Even today, the pathogenesis of IgG4-related disease is very poorly understood, although some researchers suggest that it is consistent with autoimmune and/or allergic disorder [14–16]. IgG4-related disease was first reported by Hamano et al. in 2001 as sclerosing pancreatitis with a high serum IgG4 concentration [17]. Since then, IgG4-related disease has been described in virtually every organ: biliary tree, salivary glands, periorbital tissues, kidneys, lungs, lymph nodes, meninges, aorta, breast, prostate, pericardium, and skin [1]. Histologically, IgG4-related disease is usually accompanied by numerous IgG4-secreting plasma cell infiltrations and destructive storiform fibrosis, resulting in solid mass formation that is often clinically and radiologically misdiagnosed as malignant neoplasm [18]. Obliterative phlebitis and increased serum IgG4 levels are often but not always identified in these patients [18, 19].

IgG4-related thyroiditis has not been definitely recognized as a separate category yet. It has been widely accepted that some cases of Hashimoto and Riedel thyroiditis can be recategorized into IgG4-related diseases because of lymphocyte infiltration rich in IgG4-secreting plasma cells and marked storiform fibrosis [9, 10, 20]. Recently, Kottahachchi et al. claimed that four inflammatory disorders were comparable with IgG4-related disease in thyroid: Riedel thyroiditis, fibrosing variant of Hashimoto thyroiditis, IgG4-related Hashimoto thyroiditis, and Graves disease with elevated IgG4 level [21]. However, unlike IgG4-related diseases of the other organs, the thyroid affected in IgG4-related thyroiditis is entirely enlarged, similar to that in non-IgG4-related conventional thyroiditis, and a localized fibrous mass is scarcely ever formed [12, 22]. In the detailed cohort study by Raess et al., the amount of infiltrating IgG4-secreting plasma cells in thyroiditis is not correlated to the histological findings (such as stromal fibrosis) typically seen in IgG4-related disease in other organs [23]. In the present case, dense stromal fibrosis was focally progressed, resulting in mass formation, on a background rich in lymphocyte and plasma cell infiltrations reminiscent of lymphocytic thyroiditis. The histology is considered to differ from the other manifestations of IgG4-related thyroiditis that are already defined.

In addition, the mass was preoperatively misinterpreted as thyroidal carcinoma in the present case. It is well known that Riedel thyroiditis is also frequently misdiagnosed because fibrosis, often extending beyond the thyroid, is marked so that the thyroid seems to be entirely occupied by thyroidal cancer [12]. Meanwhile, in this case, the mass was formed inside the thyroid, and extrathyroidal extension was not observed, unlike that seen in Riedel thyroiditis. Therefore, mass-forming thyroiditis may be another pattern of IgG4-related thyroiditis that clinically and radiologically differs from Hashimoto and Riedel thyroiditis and Graves disease, as well as from thyroidal carcinomas.

IgG4-related thyroiditis, like IgG4-related disease of the other organs, can be divided into two categories: the organ-specific type and the systemic type. Li et al. suggest that IgG4-related Hashimoto thyroiditis is compatible with the thyroid-specific type, whereas IgG4-related Riedel thyroiditis is a manifestation of the systemic type of IgG4-related disease [11]. In the present case, IgG4-related inflammation spread not only into the thyroid but also into the perithyroidal lymph nodes; this finding indicates the supervenience of IgG4-related lymphadenopathy because IgG4-secreting plasma cells are scarcely observed in lymph nodes of the patients with non-IgG4-related lymphadenopathies [24]. However, lymphadenopathy was observed not systemically but just regionally. Only very few cases of autoimmune pancreatitis and IgG4-related gastritis with regional lymphadenopathy have been reported [25, 26], and there have been no reports of cases accompanied by IgG4-related thyroiditis. Therefore, IgG4-related regional lymphadenopathy could be misdiagnosed as metastatic thyroidal carcinoma, as in the present case. The clinical and pathological manifestation of IgG4-related regional lymphadenopathy is poorly understood because regional lymph nodes are rarely dissected and examined. Some reports suggest that organ-specific IgG4-related diseases can be treated by surgical resection [27]; however, remaining regional lesions may be associated with the prognosis. More cases with regional lymphadenopathy are required to provide new insights into the pathoetiology, clinical behavior, and prognostic factors of IgG4-related thyroiditis.

## Conclusions

It has been widely accepted that in IgG4-related thyroiditis, the thyroid is entirely enlarged and diffusely affected, which is similar to that in conventional Hashimoto and Riedel thyroiditis and Graves disease, but rarely ever accompanied by pseudoneoplastic mass formation as in IgG4-related disease in the other organs. Mass-forming thyroiditis may be distinguishable from the other manifestations of IgG4-related thyroiditis that are already defined, and it can be clinically and radiologically misinterpreted as thyroidal carcinoma because it forms a somewhat circumscribed intrathyroidal mass. In addition, regional, not systemic, IgG4-related lymphadenopathy, also possibly misdiagnosed as metastatic thyroidal carcinoma, may be a newly recognized manifestation of IgG4-related thyroiditis.

## Abbreviation

Ig: Immunoglobulin
